# A Common Genetic Variant (97906C>A) of DAB2IP/AIP1 Is Associated with an Increased Risk and Early Onset of Lung Cancer in Chinese Males

**DOI:** 10.1371/journal.pone.0026944

**Published:** 2011-10-26

**Authors:** Lei Yang, Yinyan Li, Xiaoxuan Ling, Lin Liu, Bin Liu, Kevin Xu, Xiaonong Bin, Weidong Ji, Jiachun Lu

**Affiliations:** 1 The Institute for Chemical Carcinogenesis, The State Key Lab of Respiratory Disease, Guangzhou Medical University, Guangzhou, People's Republic of China; 2 Department of Pathology, Yale University School of Medicine, New Haven, Connecticut, United States of America; Univesity of Texas Southwestern Medical Center at Dallas, United States of America

## Abstract

DOC-2/DAB2 interactive protein (DAB2IP) is a novel identified tumor suppressor gene that inhibits cell growth and facilitates cell apoptosis. One genetic variant in *DAB2IP* gene was reported to be associated with an increased risk of aggressive prostate cancer recently. Since DAB2IP involves in the development of lung cancer and low expression of DAB2IP are observed in lung cancer, we hypothesized that the variations in *DAB2IP* gene can increase the genetic susceptibility to lung cancer. In a case-control study of 1056 lung cancer cases and 1056 sex and age frequency-matched cancer-free controls, we investigated the association between two common polymorphisms in *DAB2IP* gene (−1420T>G, rs7042542; 97906C>A, rs1571801) and the risk of lung cancer. We found that compared with the 97906CC genotypes, carriers of variant genotypes (97906AC+AA) had a significant increased risk of lung cancer (adjusted odds ratio [OR] = 1.33, 95%CI = 1.04–1.70, *P* = 0.023) and the number of variant (risk) allele worked in a dose-response manner (*P*_trend_ = 0.0158). Further stratification analysis showed that the risk association was more pronounced in subjects aged less than 60 years old, males, non-smokers, non-drinkers, overweight groups and in those with family cancer history in first or second-degree relatives, and the 97906A interacted with overweight on lung cancer risk. We further found the number of risk alleles (97906A allele) were negatively correlated with early diagnosis age of lung cancer in male patients (*P* = 0.003). However, no significant association was observed on the −1420T>G polymorphism. Our data suggested that the 97906A variant genotypes are associated with the increased risk and early onset of lung cancer, particularly in males.

## Introduction

Lung cancer is the most common type of cancer and the number one cause of death among tumor types in the world [1]. In the United States, it was estimated that there would be 222,520 new lung cancer incidences and 157,300 lung cancer deaths in 2010 [1]. Data from the national death survey and cancer registration of China mainland also showed that lung cancer was the leading cause of death in most of cities in China [2]. In Guangzhou, the incidence rate of lung cancer was 61.18/10^5^ in males, and 32.56/10^5^ in females in 2004, accordingly, with a death rate of 59.37/10^5^ in males, and 27.82/10^5^ in females [2]. All these indicated that the burdens of lung cancer are serious all over the world, especially in males.

Epidemiologic studies have showed that the most etiological factors of lung cancer are from environment, including cigarette smoking, occupational exposure to polycyclic aromatic hydrocarbons, asbestos, metals, and welding fumes, air pollution, and malnutrition or diets [3]. However, only a small portion of individuals who expose to hazardous environmental factors developed lung cancer disease, which suggests that factors other than environment, such as the host (e.g., chronic lung diseases) or genetic factors (e.g., gene variation) may predispose individuals to develop lung cancer [4]. Recently, a meta-analysis including results from 41 studies reported that the lung cancer family history was a risk factor of lung cancer [5]. These demonstrated that the genetic factor plays a role in the development of human lung cancer.

*DAB2IP*, the coding gene of DOC-2/DAB2IP protein, also known as apoptosis signal- regulating kinase 1–interacting protein-1 (AIP1), is a novel member of the RAS GTPase- activating gene family. DAB2IP, not only directly activates TNF- induced ASK1-JNK cell apoptotic signaling and inhibits TNF- induced IKK- NF-κB cell growth signaling [6]–[8], it also suppresses the phosphatidylinositol 3-kinase (PI3K)-Akt pathway and thus leads to cell apoptosis [9] and negatively regulate RAS-mediated cell survival signal via its interaction with DAB2 [10]. Moreover, study also revealed a function of DAB2IP in modulating epithelial-to-mesenchymal transition and cancer metastasis [11]. Due to the aberrant methylation in the *DAB2IP* promoter region, low expression of DAB2IP was found in several types of cancer including lung cancer, and increased level of DAB2IP can suppress cancer growth [12]–[17]. DAB2IP knockout mice showed that deficiency of DAB2IP protein can strongly augment epithelial cell migration and inflammatory angiogenesis [18]. All the data suggests that *DAB2IP* may function as a tumor suppressor gene.

*DAB2IP* gene locates at chromosome 9q33.1–33.3. It is high polymorphic, 1457 polymorphisms and two common different transcripts have been reported in this gene. Recently, one study reported that a genetic polymorphism (rs1571801) in intron 1 of *DAB2IP* gene was significantly associated with increased risk of aggressive prostate cancer both in African American and European population [19]. Because most of the lung cancer patients are males and DAB2IP deficient was also founded in lung cancer [15], it is likely that the genetic variation in *DAB2IP* gene may contribute to increase the risk of lung cancer as it did in prostate cancer. Variations in the promoter of this gene may affect gene's functions. Taken these together, we hypothesized that the genetic variations in *DAB2IP* gene and their possible interactions with environmental factors are associated with the risk of lung cancer.

In this hospital-based case-control study including 1056 lung cancer patients and 1056 sex and age frequency-matched genetic unrelated cancer-free controls, we genotyped two common polymorphisms in *DAB2IP* gene (−1420T>G, rs7042542, in promoter region; 97906C>A, rs1571801, in intron 1) and investigated their association with the risk of lung cancer.

## Results

### Subjects' demographics

The distributions of demographic variables and risk factors of the 1056 cases and the 1056 cancer-free controls have been described elsewhere [20] and these variables were further adjusted for the multivariate logistic regression model to control possible confounding of the main effects of the studied SNP and used in the later stratification and gene- environment interaction analysis. In addition, of the 1056 cases, there were 683(64.7%) cases of cantoneses, 90(8.5%) teocheweses, 198(18.8%) hakkases and 85(8.0%) subjects from other provinces. of the 1056 controls, there were 700(66.2%) cantoneses, 81(7.7%) teocheweses, 195(18.5%) hakkases and 80(7.6%) subjects from other provinces. The difference in distributions of sub-nation between cases and controls were not statistically significant (*P* = 0.8538) and this was used in the later and gene-environment interaction stratification analysis (Table 1).

**Table 1:** Frequency distributions of selected variables in LC patients and cancer-free controls.

Variables	Patients (n = 1056)		Controls (n = 1056)		*P* a
	*N*	%	*N*	%	
Age (years)					0.9306
≤60	536	50.8	534	50.6	
>60	520	49.2	522	49.4	
Sex					1.000
Male	746	70.6	746	70.6	
Female	310	29.4	310	29.4	
Smoking status					0.0283
Current	394	37.3	366	34.7	
Former	207	19.6	176	16.7	
Never	455	43.1	514	48.6	
Alcohol drinking					0.0429
Current	165	15.6	186	17.6	
Former	64	6.1	41	3.9	
Never	827	78.3	829	78.5	
Family history of cancer					0.9417
Yes	104	9.9	103	9.8	
No	952	90.1	953	90.2	
BMI(kg/m^2^)					<.0001
<18	134	12.7	51	4.8	
18–25	820	77.6	702	66.5	
>25	102	9.7	303	28.7	
Sub-ethic					0.8538
Cantonese	683	64.7%	700	66.2%	
Teochewese	90	8.5%	81	7.7%	
Hakkas	198	18.8%	195	18.5%	
Other provinces	85	8.0%	80	7.6%	

a *P* values for a two-sided χ^2^ test.

### The distributions of *DAB2IP* genotypes and the risk of lung cancer

The distributions of the two SNPs genotypes among the controls and cases, and the adjusted odds ratios associated with lung cancer are summarized in Table 2. Both genotype frequencies in control population were in agreement with those predicted under Hardy-Weinberg equilibrium (*P* = 0.454 for −1420T>G; *P* = 0.113 for 97906C>A). Genetic linkage disequilibrium between the −1420T>G locus and 97906C>A locus did not occur as the LD analysis in controls shown (D′ = 0.015 and r^2^ = 0.001), suggesting each may have an independent effect on risk of lung cancer.

**Table 2:** Distribution of genotypes in *DAB2IP* and results of logistic regression analysis for associations with risk of LC.

Genotypes	Patients	Controls^a^	*P* b	CrudeOR (95% CI)	AdjustedOR (95% CI)^c^
	*n* (%)	*n* (%)			
Total no. of subjects	1056	1056			
Total no. of alleles	2122	2122			
rs7042542(−1420T>G)			0.5263		
TT	830(78.6)	847(80.2)		1.00 (ref.)	1.00 (ref.)
GT	219(20.7)	200(18.9)		1.12(0.90–1.38)	1.09(0.87–1.37)
GG	7(0.7)	9(0.9)		0.80(0.30–2.14)	0.81(0.29–2.22)
Trend test P value				0.4459	0.5783
GT+GG	224(21.4)	209(19.8)		1.10(0.89–1.36)	1.08(0.87–1.35)
G allele	0.110	0.103	0.4549		
rs1571801(97906C>A)			**0.0381**		
CC	870 (82.4)	912 (86.4)		1.00 (ref.)	1.00 (ref.)
CA	172 (16.3)	135 (12.8)		**1.34 (1.05–1.70)**	**1.31 (1.01–1.68)**
AA	14 (1.3)	9 (0.8)		1.63 (0.70–3.79)	1.90 (0.80–4.53)
Trend test P value				**0.0111**	**0.0158**
CA+AA	186 (17.6)	144 (13.6)		**1.35(1.07–1.72)**	**1.33 (1.04–1.70)**
A allele	0.095	0.072	**0.0090**		

a The observed genotype frequencies among the control subjects were in agreement with the Hardy-Weinberg equilibrium (*p* ^2^+2*pq*+*q* ^2^ = 1) (*χ^2^* = 2.51, P = 0.113 for 97906C>A; *χ^2^* = 0.56, P = 0.454 for −1420T>G).

b Adjusted in a logistic regression model that included age, sex, smoking status, alcohol use, BMI, family history of cancer.

c A two-sided χ^2^ tests for differences in distribution of genotype frequencies between cases and controls.

As shown in Table 2, Allele frequency of 97906C>A SNP was shown significant differences between the cases and controls (*P* = 0.0381). Multivariate logistic regression analysis showed that after adjusting for confounding factors, compared with CC genotypes, CA had a significantly increased risk of lung cancer (adjusted odds ratios [OR] = 1.31; 95% CI = 1.01–1.68; *P* = 0.0436), the AA homozygote had a non-significantly increased risk of lung cancer (adjusted OR = 1.90; 95%CI = 0.80–4.53; *P* = 0.1438) because of its rare frequency, but there was a significant trend for an allele dose effect on risk of lung cancer (*P*_trend_ = 0.0158). Under the co-dominant model of inheritance, the carriers of A allele (i.e., CA/AA genotypes) had a 0.33-fold increased risk of lung cancer (95%CI  = 1.04–1.70; *P* = 0.023). However, for the −1420T>G SNP, the genotype and allele frequencies did not differ significantly between the cases and the controls (*P* = 0.5263 and 0.4549, respectively). Consistently, there was no significant association between this SNP and risk of lung cancer.

### Stratification analysis of *DAB2IP* 97906C>A genotypes and risk of lung cancer

We further evaluated the associations of the 97906CA/AA variant genotypes with lung cancer risk stratified by selected variables. As shown in Table 3, compared with the common wild-type homozygous genotype, the detrimental genotypes 97906CA/AA were more pronounced in males (adjust OR = 1.42, 95% CI = 1.06–1.89), in younger individuals (adjust OR = 1.51, 95% CI = 1.06–2.14), in non-smokers(adjust OR = 1.43, 95% CI = 1.00–2.10), in nondrinkers (adjust OR = 1.45, 95% CI = 1.10–1.92), in overweight groups (adjust OR = 2.33, 95% CI = 1.30–4.16) and in those with family cancer history in first or second-degree relatives(adjust OR = 2.55, 95% CI = 1.04–6.25). Whereas, no significant association between the −1420T>G polymorphism and the risk of lung cancer was found in any of the subgroups (data not shown).

**Table 3:** Stratification analysis of the *DAB2IP* 97906C>A genotypes by selected variables in LC patients and controls.

	Patients (*n* = 1056)		Controls (*n* = 1056)		Crude OR(95% CI)	Adjusted OR(95% CI)^a^	
	CC*n* (%)	CA+AA*n* (%)	CC*n* (%)	CA+AA*n* (%)	CA+AA vs CC	CA+AA vs CC	*P*_interaction_ b
Age (years)							
≤60	442 (82.5)	94(17.5)	467(87.5)	67(12.5)	**1.48 (1.06–2.08)**	**1.51(1.06–2.14)**	0.4357
>60	428 (82.3)	92(17.7)	445 (85.2)	77(14.8)	1.24 (0.90–1.73)	1.16(0.82–1.65)	
Sex							
Male	612 (82.0)	134 (18.0)	642 (86.1)	104 (13.9)	**1.35 (1.02–1.79)**	**1.42 (1.06–1.89)**	0.4145
Female	258 (83.2)	52 (16.8)	270 (87.1)	40 (12.9)	1.36 (0.87–2.13)	1.12 (0.70–1.80)	
Smoking status							
Never	377(82.9)	78(17.1)	456(88.7)	58(11.3)	**1.63(1.13–2.35)**	**1.43(1.11–2.10)**	0.9460
Ever	493(82.0)	108(18.0)	456(84.1)	86(15.9)	1.16(0.85–1.59)	1.26(0.91–1.75)	
Drinking status							
Never	682(82.5)	145(17.5)	723(87.2)	106(12.8)	**1.45(1.11–1.90)**	**1.45(1.10–1.92)**	0.4018
Ever	188(82.1)	41(17.9)	189(83.3)	38(16.7)	1.09(0.67–1.76)	1.03(0.62–1.74)	
BMI(kg/m^2^)							
<18	108(80.6)	26(19.4)	40(78.4)	11(21.6)	0.88(0.40–1.93)	0.79(0.34–1.83)	**0.0348**
18–25	685(83.5)	135(16.5)	607(86.5)	95(13.5)	1.26(0.95–1.67)	1.25(0.94–1.66)	
>25	77(75.5)	25(24.5)	265(87.5)	38(12.5)	**2.26(1.29–3.99)**	**2.33(1.30–4.16)**	
Family history of cancer							
YES	84(80.8)	20(19.2)	94(91.3)	9(8.7)	**2.49(1.07–5.76)**	**2.55(1.04–6.25)**	0.1371
NO	786(82.6)	166(17.4)	818(85.8)	135(14.2)	1.28(1.00–1.64)	1.26(0.98–1.64)	
Sub–ethic							0.6660^c^
Cantonese	569(83.3)	114(16.7)	603(86.1)	97(13.9)	1.34(0.64–2.82)	1.35(0.61–3.09)	
Teochewese	69(76.7)	21(23.3)	66(81.5)	15(18.5)	1.53(0.87–2.68)	1.70(0.94–3.09)	
Hakkas	163(82.3)	35(17.7)	171(87.7)	24(12.3)	1.25(0.93–1.67)	1.24(0.91–1.69)	
Other provinces	69(81.2)	16(18.8)	72(90.0)	8(10.0)	2.09(0.84–5.19)	2.05(0.78–5.42)	
Histological types							
Adenocarcinoma	303(82.1)	66(17.9)	912(86.4)	144(13.6)	1.38(1.01–1.90)	1.26(0.79–1.89)	0.5680^c^
Squamous cell carcinoma	312(81.2)	72(18.8)			1.46(1.07–1.99)	1.11(0.72–1.71)	
Large cell carcinoma	37(86.0)	6(14.0)			1.03(0.43–2.48)	1.39(0.46–4.33)	
Small cell lung cancer	107(83.6)	21(16.4)			1.24(0.75–2.05)	0.72(0.32–1.61)	
Other carcinomas^c^	111(84.1)	21(15.9)			1.20(0.73–1.97)	0.69(0.32–1.50)	

a ORs were adjusted for age, sex, smoking status, and alcohol use, BMI, family history of cancer in a logistic regression models.

b *P* value of a test the multiplicative interaction between 97906C>A and selected variables on cancer risk were calculated using standard unconditional logistic regression models.

c *P* value of the test for homogeneity between stratum-related ORs for *DAB2IP* (97906CA+AA versus CC genotypes).

Furthermore, we assessed possible interactions (or effect modifications) between the polymorphism and age, sex, smoking, drinking, family history of cancer, and BMI groups on cancer risk. While fitting multiplicative models, we only found that the 97906A variant genotypes interacted with BMI on increasing cancer risk (interaction test *P* = 0.0348). We further analyzed the possible interactions with the Multifactor Dimensionality Reduction (MDR) software, and found that there were interactions of the 97906A variant genotypes, BMI and smoking on increasing cancer risk (interaction test *P* = 0.0003). These results suggest a possibility of interaction between 97906A variant genotypes and overweight on lung cancer risk.

### Age at onset of lung cancer and variants of *DAB2IP*

**Figure 1:**
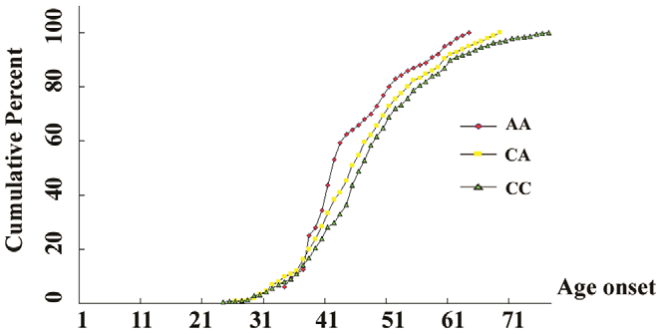
Age at onset of lung cancer and variants of *DAB2IP* gene.

Because an early age at onset of cancer is a feature of genetic susceptibility to the disease, we analyzed the relationship between age at onset of lung cancer patients and the genotypes of *DAB2IP*, A Pearson correlation analysis showed that age at onset decreased significantly as the number of risk alleles increased in males (r = −0.11, *P* = 0.0026), but not in females (r = −0.06, *P* = 0.2923). We further assigned those 746 male patients to one of three groups on the basis of the number of risk alleles, and found that the age at onset (mean ± SD) was 61.1±11.7 years in 610 patients with zero risk allele (i.e., CC genotypes), it was significantly lower in 124 patients with one risk allele (CA genotypes) (58.8±11.2); and it was further decreased in 12 patients with two risk alleles (AA genotypes) (55.4±9.5). The carriers of 97906CA or AA genotypes were 2.3 years or 5.7 years younger than that of CC genotypes. (ANOVA test *P* = 0.0386) (Fig. 1). We further performed Cox regression analysis with and without adjustments for sex, age, smoke status, drink status and BMI, and found that the genetic variant 97906A (97906CA and AA) of *DAB2IP* was an independent factor for the early onset of lung cancer patients (*P* = 0.033, *P*_ad_ = 0.031).

### Association between the 97906C>A genotype and *DAB2IP* mRNA levels

**Figure 2:**
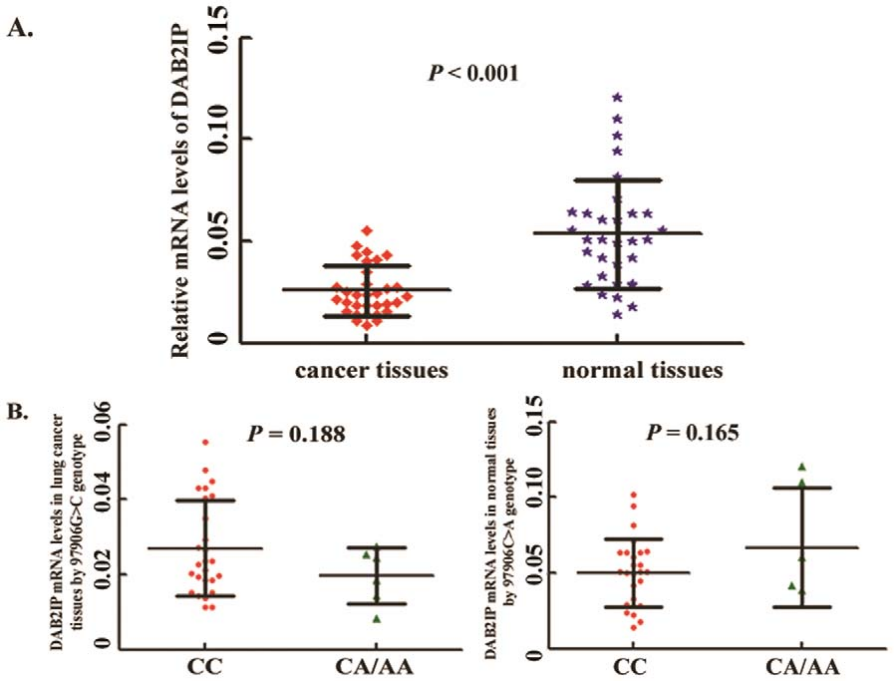
Association of the 97906C>A genotype and *DAB2IP* expressions. **A,** Relative mRNA levels of the *DAB2IP* expressions in lung cancer tissues compared to their adjacent normal lung tissues; **B,** Relative mRNA level of the *DAB2IP* expression by the 97906C>A genotype in lung cancer tissues. **C,** Relative mRNA level of the *DAB2IP* expression by the 97906C>A genotype in normal tissues. No significant association was observed between the 97906C>A genotype and *DAB2IP* mRNA levels neither in cancer tissues nor in normal tissues Columns, mean from three independent experiments; bars, SD; and Student's t test was used to test the differences in the expression levels of different constructs.

As shown in Figure 2A, the mRNA levels of *DAB2IP* in 32 lung cancer tissues were significantly lower than their adjacent normal tissues (*P*<0.001). However, the differences of DAB2IP mRNA expression among 97906C>A genotypes were not significant in neither tumor tissues (*P = *0.188) nor normal tissues (*P = *0.165), as shown in Figure 2B and 2C. It suggested that the SNP 97906C>A may have no significant impact on this gene's expression.

### Detection of DAB2IP/MLL fusion gene

In genotyping analysis of 97906C>A genotypes in those 82 lung cancer tissues and their adjacent normal tissues, we found that of cancer tissues 19 cases were carriers of CA, 2 cases were AA, of the adjacent normal tissues 17 cases were carriers of CA and one case was AA. We amplified the fragment of DAB2IP/MLL fusion gene with PCR method as von Bergh AR described for all of those 82 lung cancer tissues and their adjacent normal tissues. However, we failed to detect *AB2IP/MLL* fusion gene.

## Discussion

In this lung cancer case–control study in a southern Chinese population, we investigated association between two common variants of *DAB2IP* and the risk of lung cancer with a relatively large sample size of 1056 lung cancer patients and 1056 cancer-free controls. Significant association was observed between the 97906C>A polymorphism and lung cancer risk. The 97906A variant genotypes were found to be associated with increased risk, especially in the subgroups of subjects younger than 60 years, males, never smokers, never drinkers, overweighter or obesities, and those with family cancer history. Furthermore, the increased number of risk alleles was associated with more early age at onset of lung cancer. However, for the polymorphism −1420T>G, no evidence was found for any association between this SNP and lung cancer risk. To the best of our knowledge, this is the first genotype association study of *DAB2IP* SNPs and lung cancer risk.

One study found the intron1 genetic variant 97906A in *DAB2IP* was associated with an increased risk of aggressive prostate cancer [19]. Because of the critical role of DAB2IP in the etiology of lung cancer, here we found that the 97906C>A SNP was also associated with an increased risk of lung cancer in males; it suggests that the 97906A variants may play an important role in etiologic of lung cancer. We had performed bioinformatics' analysis with VISTA tools (http://genome.lbl.gov/vista/index.shtml) by comparing the genomic structures of *DAB2IP* gene among human (GeneID: 153090) and mouse (GeneID: 69601), rat (GeneID: 192126), dog (GeneID: 491356), cow(GeneID: 541268), we found that the 97906C>A locus locates in evolutionarily conserved regions (ECRs) which do not changed through evolution and served as functional cis-acting elements [21]. We further analyzed the possible genetically statistical similar SNPs to 97906C>A in a 5 Mb region on chromosome 9 via the HapMap SNP database (release #36, http://www.hapmap.org/, November 9, 2009) genotyped in the CHB trios with the ssSNPer web interface (http://gump.qimr.edu.au/general/daleN/ssSNPer/). Ina region from 123228424 bp upstream to 123728423 bp downstream of 97906C>A, we also found one intron 1 polymorphisms rs1984038 which was 3184 bp upstream of 97906C>A was in completely LD (D′ = 1.00, r^2^ = 1.00) with 97906C>A [22], and it was also belong to the ECRs. However, we neither found any significant effect of 97906C>A SNP on the mRNA expressions of DAB2IP gene in lung cancer tissues and their adjacent normal tissues; and we failed to detect the possible impact of the SNP on DAB2IP/MLL fusion gene in cancer tissues. Taking these evidences together, it demonstrated that 97906C>A is a conserved genetic marker.

In the stratification analysis, we found that the variant genotypes (97906CA+97906AA) were more pronounced in male, younger, never smokers, nondrinkers, overweight groups and those with family history of cancer. Rigorous environmental factors such as smoking are risk of lung cancer and gene-environment interaction is well known involved in the etiology of lung cancer [23], but here we found that the 97906A variant may influence the development of lung cancer mostly due to autochthonous inherited effect. As shown, 97906C>A is more significant in young individuals, in nonsmokers and in nondrinkers. Constantly those with family history of cancer in any first- or second - degree relatives were more tend to develop lung cancer due to the genetic background high 97906A frequency. In addition, we observed consistent evidence for a statistically significant interaction between the 97906A and overweight. We supposed androgens may act as an intermediary for the close correlation between androgens and obesity [24] and androgens, function on the expression of DAB2IP by suppressing EZH2 expression [25]. Further studies are needed to reveal the mechanism as accumulated studies have verified that overweight increases the risk of human cancer, including lung cancer [26].

Interestingly, we also found that the 97906A variant genotypes were associated with an early onset age of lung cancer in male patients in an allele dose-effect manner. DAB2IP may relate to the pathogenesis of lung cancer as study have shown that in prostate cancer DAB2IP influenced the repair effectiveness of DNA double-strand breaks induced by medical exposure to ionizing radiation which is a risk factor of lung cancer [27]. Thus, our findings demonstrated the 97906C>A polymorphism may be a conserved genetic marker to predict the risk of lung cancer and further early onset of lung cancer in males.

Since the present study was a hospital-based case-control study, restricted to Chinese Han population, it may have some limitations. However, the genotype frequency distributions (i.e., −1420G, 97906A alleles were 0.103, 0.072, respectively) in the control subjects of our study were similar to those reported for Chinese population including in HapMap database (i.e., −1420G, 97906A alleles were 0.114, 0.067, respectively). Moreover, the genotype frequencies among controls could fit the Hardy–Weinberg law also suggested the randomness of subject selection. Von Bergh AR et al. reported that the exon 2 of *DAB2IP* gene was fused with *MLL* gene in an acute myeloid leukemia patient. However, we failed to observe the *DAB2IP/MLL* fusion gene in 82 cases of lung cancer tissues and their adjacent normal tissues, let alone the possible effect of 97906C>A polymorphism on *DAB2IP/MLL* fusion gene. Due to the technological limitations, we did not yet perform any other functional assay to identify whether 97906C>A locus is in an intronic enhancer or a splicing-related site. In this association study, we achieved an 80.2% study power to detect crude OR of 1.35 for the 97906CA+AA genotypes (which occurred at a frequency of 13.6% in the controls) compared with the 97906CC genotype. Therefore, it appears to be that the 97906CA+AA genotypes associated with an increased risk of lung cancer is unlikely to have been obtained by chance.

In conclusion, in this hospital-based case-control study of lung cancer, we found that the 97906A genetic variants of *DAB2IP* gene was associated with early onset age and an increased risk of lung cancer in males, especially in the subgroups of subjects younger than 60 years, male, never drinkers, obesities, and those with family cancer history. To the best of our knowledge, this is the first study of 97906A genetic variants in *DAB2IP* gene and lung cancer susceptibility. Larger, preferably population-based case-control studies, as well as well-designed mechanistic studies, are warranted to validate our findings.

## Materials and Methods

### Study subjects and sample collection

Study subjects of 1056 lung cancer patients and 1056 cancer-free controls were recruited as previously described [20]. Briefly, all histopathologically confirmed patients with primary lung cancer were consecutively recruited at the urban hospitals and at the suburb of Guangzhou city from March 2007 to March 2009. Those with second lung tumors, or primary tumors upper tracheal bifurcation were excluded. 1056 cancer-free controls that were frequency-matched to the cases on age (±1 years) and sex were selected randomly from about 10,000 individuals who participated in the healthy checkup programs in the community health stations conducted in Guangzhou City. All subjects were genetically-unrelated ethnic Han Chinese and were from Guangzhou City and surrounding regions in Southern China. None had blood transfusions in last 6 months. A simple questionnaire was used to collect data on demographic characteristics, including smoking status, alcohol use and other factors including sex, age, BMI with signed consent form both in cases and controls. The information of the sub-ethnic (i.e., Cantonese, Teochewese, Hakkas, and Han ethnic from other provinces outside Guangdong) was also collected during interviews. All participants recruited in our study had signed a consent form before the questionnaire interview and our study was approved by the Institutional Review Boards of Guangzhou Medical University.

### SNP selection

We used the TSSW program (http://www.softberry.ru/berry.phtml) to predict promoter region and found that the 4000 bp 5′-upstream region of *DAB2IP* gene are potential promoter region. Based on the HapMap database (HapMap Data Rel 27 PhaseII +III, Feb 09, on NCBI B36 assembly, dbSNP b126), we found that there were nine common SNPs (i.e., with minor allele frequency ≥5%) within the promoter region of *DAB2IP* gene in Chinese Han Beijing population. We further selected the tagSNPs based on the pair-wise LD analysis (r^2^ threshold = 0.8) with Haploview 4.2 software, and found that the polymorphism rs7042542 (−1420T>G) was a tagSNP which representing the genetic information of other SNPs (Figure S1). In addition, adding the rs1571801 (97906C>A) SNP within the intron 1 of *DAB2IP* gene that had been reported to be significantly associated with increased risk of aggressive prostate cancer as two GWAS studies shown [19], we genotyped this two SNPs (−1420T>G, and 97906C>A) in our study (Figure S2).

### Genotyping analysis

Genomic DNA was extracted from 1 ml of a leukocyte cell pellet which was obtained from the buffy coat by centrifugation of 5 mL of peripheral blood, using the DNA Blood Mini Kit (Qiagen, Valencia, A), according to the manufacturer's instructions. DNA purity and concentrations were determined by measurement of spectrophotometric absorbance at 260 nm and 280 nm.

The two SNPs were genotyped using the polymerase chain reaction-restriction fragment length polymorphism (PCR-RFLP) method. For −1420T>G, a 132 bp PCR product was amplified from genomic DNA using specific primers: 5′-CTCCTGACCT CAGGTGATC C-3′ (forward), 5′-TGGTGGGGAGGACAAAGATGCATCTGA-3′ (reverse). PCR was performed in 25 ul reaction systems. After initial denaturation at 94°C for 5 min, there were 35 cycles at 94°C for 30 s, 61°C for 30 s, and 72°C for 60 s, and then a final extension step of 72°C for 7 min. The amplified fragments were digested with MlyI (New England BioLabs) overnight at 37°C, and the products were separated in 3.5% agarose gel. The −1420GG genotype produced 2 bands (112 and 20 bp), whereas the −1420TT genotype produced a single band (132 bp), and the heterozygotes displayed all 3 bands (132, 112 and 20 bp) (Figure S1). For 97906C>A, a 98 bp PCR product was amplified from genomic DNA using specific primers: 5′-AAACAGGCATAAGG TTTTAAGT-3′ (forward), 5′-AATTTGACTTCCAAGTTGTC-3′ (reverse). Digested with Tsp5091 (New England BioLabs) overnight at 65°C, the 97906AA genotype produced 2 bands (52 and 46 bp), 97906CC genotype produced a single band (98 bp), and the heterozygotes displayed all 3 bands (98, 52 and 46 bp) (Figure S2).

The results were evaluated by two experimenters independently who were blinded to the subjects' patient or control status. We randomly selected 10% samples for each of the 2 SNPs to perform repeat assays, and the results were 100% concordant. For each target genotype of the PCR products were purified and confirmed by direct sequencing (Figure S3).

### *DAB2IP* mRNA expression analysis

We performed qRT-PCR to determine whether the 97906C>A had an effect on *DAB2IP* gene expression *in vivo*. 32 tumor tissues and their adjacent normal tissues were collected from the patients who did not receive chemotherapy and/or radiotherapy before underwent complete surgical resection of a primary lung cancer. All tumor samples were histologically confirmed and genotypes of these samples were all sequencing confirmed. Total RNA were extracted by using Trizol Reagent (Invitrogen, Inc.). An aliquot of total RNA (1 ug) was then reverse transcribed to complementary DNA by using oligo primer and SuperscriptII (Invitrogen). Relative mRNA expression levels of *DAB2IP* and an internal reference gene *β-actin* were detected on the ABI Prism 7500 sequence detection system (Applied Biosystems) based on the SYBR-Green method. The primers used for *DAB2IP* were 5′- CTG AGC GGG ATA AGT GGA TGG -3′ (forward) and 5′- AAA CAT TGT CCG TCT TGA GCT T -3′ (reverse) and for *β-actin* were 5′-GGC GGC ACC ACC ATG TAC CCT-3′ and 5′-AGG GGC CGG ACT CGT CAT ACT-3′. Method of 2^Δt^ was used to demonstrate the level of *DAB2IP* gene's expression (Figure 2). All analyses were performed in a blinded fashion with the laboratory persons unaware of genotyping data. Each assay was done in triplicate.

### Detection of *DAB2IP/MLL* fusion gene

Because von Bergh AR et al. reported that the exon 2 of *DAB2IP* gene was fused with the intron 9 of the *MLL* gene in an acute myeloid leukemia patient and thus lost its function, we performed PCR to detect the possible effect of 97906C>A polymorphism on making *DAB2IP/MLL* fusion gene. Genomic DNA was extracted from 82 lung cancer tissues and their adjacent normal tissues were collected during the surgical excision in the first, second and tumor hospitals of Guangzhou Medical College (Guangzhou, China) and stored in −80°C refrigerator. We amplified the fragment of DAB2IP/MLL fusion gene with PCR methods as von Bergh AR described [28]. The forward primer was near exon 2 of *DAB2IP* gene (5′- CATCTGATGCCGAGGCTGAA-3′), and the reverse primer was in the exon 10 of *MLL* gene (5′-GTCCACTCTG ATCCTGTGGACT-3′). The PCR products would be 700–900 bp length. The amplified fragments were cloned with the TOPO TA cloning kit (Invitrogen, Carlsbad, CA) to purify and increase the amount, and were further sequenced by the commercial company of Invitrogen with Applied Biosystems ABI 3730 DNA analyzer.

### Statistical analysis

The chi-square tests were used to assess differences in the distribution of sub-nation between cases and controls as well as the allele and genotypes. The Hardy-Weinberg equilibrium (HWE) was tested by a goodness-of-fit chi-square test to compare the expected genotype frequencies with observed genotype frequencies in cancer-free controls. The association between case-control status and each SNP, measured with the odds ratio (OR) and its corresponding 95% confidence interval [29], was estimated using an unconditional logistic regression model, with and without adjustment for age, sex, smoking status, alcohol use, BMI. Logistic regression model was also used for the trend test. In the stratification analysis, we assessed the main effect of *DAB2IP* in each subgroup and the interaction among the *DAB2IP* polymorphisms and selected variables on cancer risk by fitting a multiplicative interaction with unconditional logistic regression model; the Multifactor Dimensionality Reduction (MDR) software was used to validate the possible interaction [30]. The 2LD program and the PROC ALLELE statistical procedure in SAS/Genetics (SAS Institute Inc., Cary, NC) software were used to detect the LD of the two SNPs. Pearson correlation analyses, ANOVA tests and Cox regression models were used for analyzing the age of onset in lung cancer patients by *DAB2IP* genotypes. All statistical tests were 2-sided and P<0.05 was considered statistically significant.

## Supporting Information

Figure S1 The block and tagSNP in the promoter region of *DAB2IP* gene.
(TIF)

Figure S2 Genomic structure, locations of SNPs in the promoter and intron 1. The nucleotide 5′ of the ATG-translation initiation codon is −1, +1 corresponds to the A of the ATG-translation initiation codon in the reference sequence. −1420T>G and −1274T>C were in complete LD. Thus, two SNPs (−1420T>G, 97906C>A) were selected in our study. Bioinformatics analysis shows that 97906C>A locates in the ECR region.
(TIF)

Figure S3 *DAB2IP* −1420T>G genotyping by direct sequencing: (a) TT genotype; (b) TG genotype; (c) GG genotype; 97906C>A genotyping by direct sequencing: (a) CC genotype; (b) CA genotype; (c) AA genotype.
(TIF)
