# Supplementary figures and images for: A Common Genetic Variant (97906C>A) of DAB2IP/AIP1 Is Associated with an Increased Risk and Early Onset of Lung Cancer in Chinese Males
Source: PLoS One. 2011 Oct 26;6(10):e26944. doi: 10.1371/journal.pone.0026944 (PMC3202597; doi:10.1371/journal.pone.0026944)

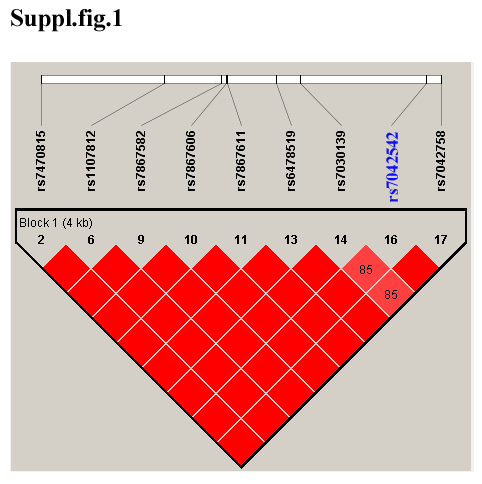

Supplement: Figure S1 — The block and tagSNP in the promoter region of DAB2IP gene. (TIF) [file pone.0026944.s001.tif]

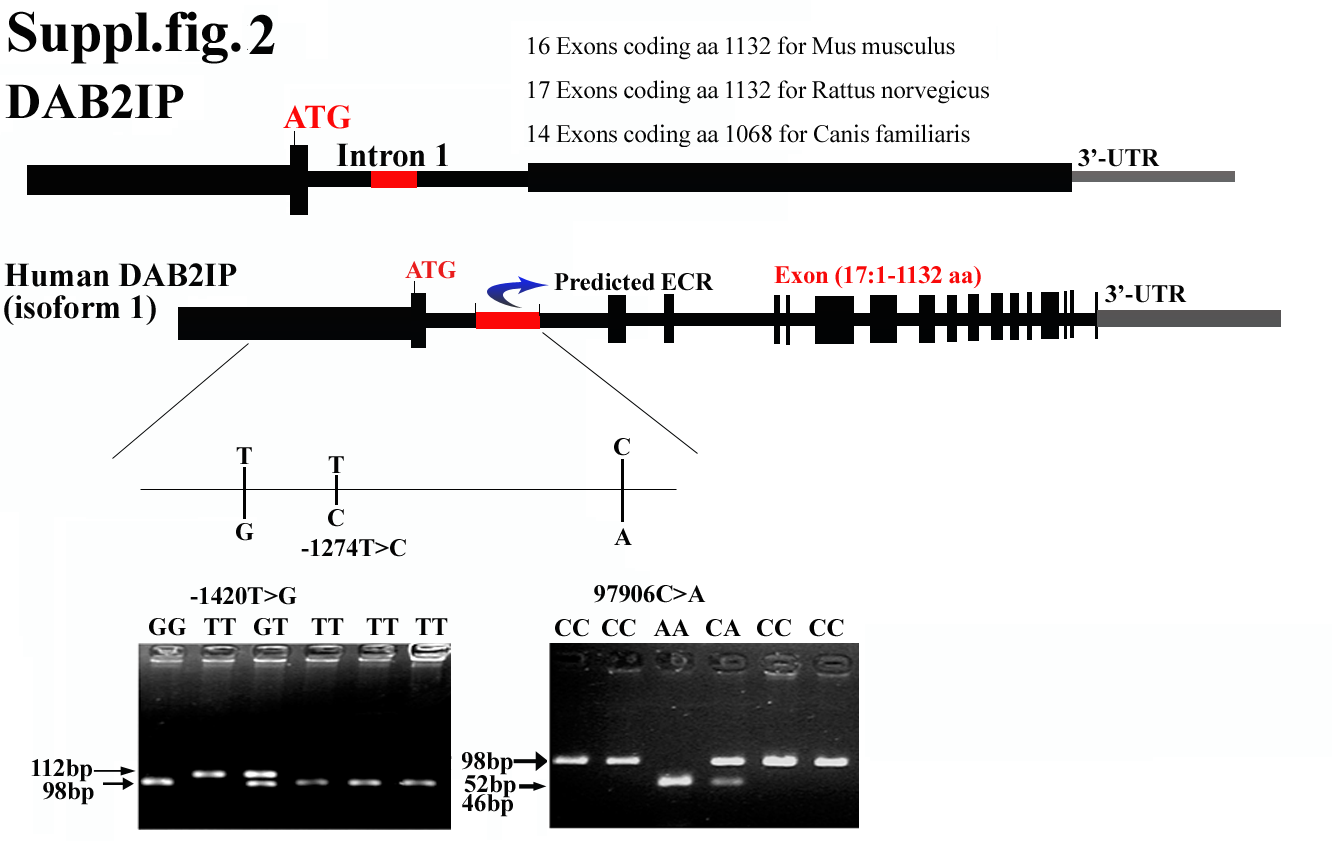

Supplement: Figure S2 — Genomic structure, locations of SNPs in the promoter and intron 1. The nucleotide 5′ of the ATG-translation initiation codon is −1, +1 corresponds to the A of the ATG-translation initiation codon in the reference sequence. −1420T>G and −1274T>C were in complete LD. Thus, two SNPs (−1420T>G, 97906C>A) were selected in our study. Bioinformatics analysis shows that 97906C>A locates in the ECR region. (TIF) [file pone.0026944.s002.tif]

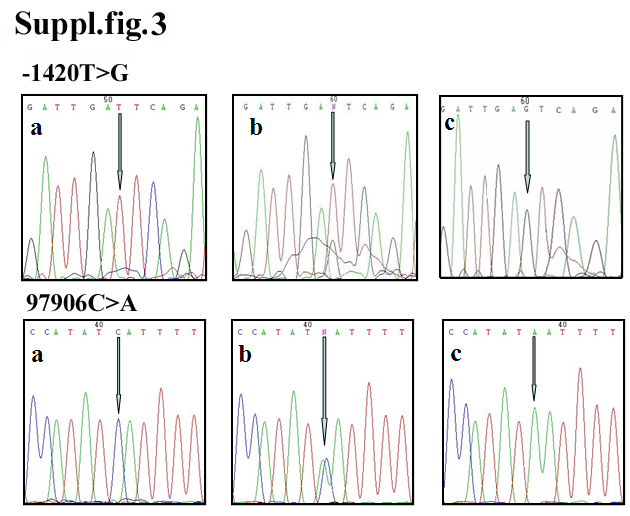

Supplement: Figure S3 — DAB2IP −1420T>G genotyping by direct sequencing: (a) TT genotype; (b) TG genotype; (c) GG genotype; 97906C>A genotyping by direct sequencing: (a) CC genotype; (b) CA genotype; (c) AA genotype. (TIF) [file pone.0026944.s003.tif]
